# Prostate Field Cancerization: Deregulated Expression of Macrophage Inhibitory Cytokine 1 (MIC-1) and Platelet Derived Growth Factor A (PDGF-A) in Tumor Adjacent Tissue

**DOI:** 10.1371/journal.pone.0119314

**Published:** 2015-03-13

**Authors:** Anna C. Jones, Kresta S. Antillon, Shannon M. Jenkins, Sara N. Janos, Heidi N. Overton, Dor S. Shoshan, Edgar G. Fischer, Kristina A. Trujillo, Marco Bisoffi

**Affiliations:** 1 University of New Mexico Health Sciences Center, Department of Biochemistry and Molecular Biology, Albuquerque, New Mexico, United States of America; 2 University of New Mexico Cancer Center, Albuquerque, New Mexico, United States of America; 3 University of New Mexico Health Sciences Center, Department of Pathology, Albuquerque, New Mexico, United States of America; 4 Chapman University, Schmid College of Science and Technology, Biochemistry and Molecular Biology/Biological Sciences, Orange, California, United States of America; Cincinnati Children’s Hospital Medical Center, UNITED STATES

## Abstract

Prostate field cancerization denotes molecular alterations in histologically normal tissues adjacent to tumors. Such alterations include deregulated protein expression, as we have previously shown for the key transcription factor early growth response 1 (EGR-1) and the lipogenic enzyme fatty acid synthase (FAS). Here we add the two secreted factors macrophage inhibitory cytokine 1 (MIC-1) and platelet derived growth factor A (PDGF-A) to the growing list of protein markers of prostate field cancerization. Expression of MIC-1 and PDGF-A was measured quantitatively by immunofluorescence and comprehensively analyzed using two methods of signal capture and several groupings of data generated in human cancerous (n = 25), histologically normal adjacent (n = 22), and disease-free (n = 6) prostate tissues. A total of 208 digitized images were analyzed. MIC-1 and PDGF-A expression in tumor tissues were elevated 7.1x to 23.4x and 1.7x to 3.7x compared to disease-free tissues, respectively (p<0.0001 to p = 0.08 and p<0.01 to p = 0.23, respectively). In support of field cancerization, MIC-1 and PDGF-A expression in adjacent tissues were elevated 7.4x to 38.4x and 1.4x to 2.7x, respectively (p<0.0001 to p<0.05 and p<0.05 to p = 0.51, respectively). Also, MIC-1 and PDGF-A expression were similar in tumor and adjacent tissues (0.3x to 1.0x; p<0.001 to p = 0.98 for MIC-1; 0.9x to 2.6x; p<0.01 to p = 1.00 for PDGF-A). All analyses indicated a high level of inter- and intra-tissue heterogeneity across all types of tissues (mean coefficient of variation of 86.0%). Our data shows that MIC-1 and PDGF-A expression is elevated in both prostate tumors and structurally intact adjacent tissues when compared to disease-free specimens, defining field cancerization. These secreted factors could promote tumorigenesis in histologically normal tissues and lead to tumor multifocality. Among several clinical applications, they could also be exploited as indicators of disease in false negative biopsies, identify areas of repeat biopsy, and add molecular information to surgical margins.

## Introduction

Adenocarcinoma of the prostate develops through increasingly malignant stages. These can include or be supported by proliferative inflammatory atrophy (PIA), a possible link between inflammatory processes and the malignant transformation of prostatic tissues [1], and by low or high grade prostatic intraepithelial neoplasia (PIN), a precursor of prostate cancer [2]. PIA and PIN are histologically evident lesions that are clearly identifiable by surgical pathologists trained in urological disorders. PIA is characterized mainly by an overall hyperchromatic appearance of glandular components with variable acinar calibers in low magnification microscopy, and the distinct presence of inflammatory cells [3]. All forms of PIN share the presence of intra-luminal proliferation of the secretory cells of the duct acinar system and abnormal cytological features, such as the ratio of nuclear-to-cytoplasmic area, chromatin content, and size of nucleoli [4]. It is conceivable that cell morphological changes leading to a histologically abnormal appearance of the glandular components of prostate tissues are preceded by a phase during which molecular alterations occur in complete absence of any cytological or histological change. This type of premalignancy is congruent with the concept of “field cancerization” or “field effect”, a term that was introduced by Daniel Slaughter in 1953 in the context of squamous oral cell carcinoma [5], and that now may exclude any cellular and histological departure from normalcy and focuses on molecular aberrations only [6]. Accordingly, a number of genetic, epigenetic, and biochemical alterations in structurally intact cells of both epithelial and stromal origin that reside in histologically normal tissues adjacent to prostate adenocarcinomas (“field cancerized” tissues) have been recently identified by us and others and postulated to be of value as clinical indicators of disease (reviewed in [7–10]). We have previously identified, among others, the key transcription factor early growth response 1 (EGR-1) and the lipogenic enzyme fatty acid synthase (FAS) as distinct markers of prostate field cancerization [11,12]. Of note, growth factors and cytokines have not been previously described in the context of field cancerization. We show here for the first time that the expression of the excreted factors macrophage inhibitory cytokine 1 (MIC-1) (also called nonsteroidal anti-inflammatory drug activated gene-1 [NAG-1], growth/differentiation factor 15 [GDF-15], and prostate derived factor [PDF]), as well as platelet derived growth factor A (PDGF-A) are deregulated in overt cancerous and tumor adjacent human tissues, when compared to donor tissues from disease-free individuals, thereby providing further evidence of prostate field cancerization.

## Materials and Methods

### Patient specimens

A cohort of 16 de-identified cases were obtained from the Cooperative Human Tissue Network (CHTN; Western Division, Nashville TN; http://www.chtn.nci.nih.gov/; 5 cases) and from the Department of Surgery, Urology Division at the University of New Mexico Hospital (UNMH) in Albuquerque NM; http://hsc.unm.edu/som/surgery/urology/; 11 cases. In agreement with all Federal, State, and University laws, the UNMH tissue samples were collected from patients undergoing prostatectomy and after written informed consent, donating approximately 100–500 mg of their tissue for molecular analyses. The Institutional Review Board of the University of New Mexico Health Sciences Center specifically approved the present study (#05–417). The cohort contained 16 prostate adenocarcinomas, of which 13 were matched to tumor-adjacent prostate tissues. The mean age of this patient cohort was 58.7 years with a range of 44–68 years. These specimens featured Gleason scores from 6 to 9 and pathological tumor node metastasis (TNM) stages (according to the American Joint Committee on Cancer) from T2c to T3b. For 3 tumor tissues, the corresponding adjacent tissues were of insufficient quality for inclusion into the final results. Six de-identified and entirely disease-free prostate specimens from autopsy cases from individuals who died due to conditions unrelated to cancer were obtained from the CHTN. The mean age of the autopsy cases was 48.7 years with a range of 26–79 years. All tissues were histologically reviewed by our collaborating surgical pathologist (E.G Fischer), especially to exclude the presence of cryptic cancer cells in the tumor adjacent prostate tissues. A human prostate tissue microarray featuring 9 histologically normal tissues matched to their corresponding tumors was purchased from Novus Biologicals (catalog #NBP2–30169; San Diego CA). The mean age of the tissue microarray cases was 64.2 years with a range of 44–70 years. These specimens featured Gleason scores from 7 to 10 and pathological TNM stages from T2c to T3b. Table 1 shows all demographic and pathological data.

**Table 1:** Demographics and clinical parameters of prostate tissues, and number of images analyzed.

Prostate tissues	Age	TNM ^1^	Gleason	Number of images analyzed			
***Disease-free (CHTN)***				**MIC-1**		**PDGF-A**	
1	26	not applicable		3		0	
2	43			3		0	
3	46			0		3	
4	79			4		2	
5	43			3		3	
6	55			3		2	
				**Total = 16**		**Total = 10**	
***Tumor and adjacent(UNMH/CHTN)***	**Age**	**TNM** ^**1**^	**Gleason**	**Tumor**	**Adjacent**	**Tumor**	**Adjacent**
1	51	n/a ^2^	7 (3+4)	3	0	0	0
2	54	T3a	7 (3+4)	3	0	0	0
3	59	T3b	9 (4+5); 6 (3+3)	3	3	3	3
4	63	T3a	6 (4+3)	5	3	2	3
5	69	T2c	7 (4+3)	3	3	6	3
6	68	T3b	8 (5+3)	4	2	3	3
7	55	T2c	8 (3+5)	6	6	9	0
8	57	T3a	7 (4+3)	3	3	3	3
9	55	T2c	8 (3+5)	0	0	3	3
10	54	T2-T3	6 (3+3)	0	0	0	6
11	54	T2c	6 (3+3)	0	0	0	5
12	64	T3b	6 (3+3)	0	0	4	4
13	62	T2c	6 (3+3)	0	9	0	9
14	62	T3b	7 (4+3)	4	5	3	3
15	44	T2c	6 (3+3)	0	0	3	0
16	68	T3a	7 (3+4)	3	6	3	0
				**Total = 37**	**Total = 40**	**Total = 42**	**Total = 45**
***Tumor and adjacent(TMA)***	**Age**	**TNM** ^**1**^	**Gleason**	**Tumor**	**Adjacent**		
1	69	T2c	7	1	1	not done	
2	62	T2c	7	1	1		
3	66	T3a	7	1	1		
4	65	T3b	9	1	1		
5	69	T3a	7	1	1		
6	70	T3a	7	1	1		
7	70	T2c	7	1	1		
8	63	T3b	10	1	1		
9	44	T3b	7	1	1		
				**Total = 9**	**Total = 9**		

A total of 25 adenocarcinomas, 22 tumor adjacent (18 matched), and 6 disease-free tissues were analyzed. In total, 208 images were queried (numbers for each case and marker are indicated). Specimens were collected at the University of New Mexico Hospital (UNMH, Albuquerque NM), obtained from the Cooperative Human Tissue Network (CHTN; Nashville TN), or featured on tissue microarrays (TMA) from Novus Biologicals (San Diego CA).

^1^ Tumor Nodes Metastasis (TNM) pathological stage was assigned using criteria published by the American Joint Committee on Cancer (http://www.cancerstaging.org/index.html).

^2^ n/a, not available.

### Quantitative immunofluorescence

Quantitative immunofluorescence was performed as described in our previous work [12]. Briefly, paraffin-embedded prostate tissue sections were deparaffinized with xylene and rehydrated with decreasing concentrations of ethanol. Antigen retrieval was performed in boiling 10mM Tris, 1mM EDTA, 0.05% Tween 20, pH 9.0 (by HCl) for 20 minutes, washed briefly in tap water, followed by gentle agitation in Tris buffered saline (TBS; 50mM Tris, 150mM NaCl, pH 7.6 by HCl) containing 0.025% Triton X-100 (TBST). Tissues were blocked in 10% normal goat serum (sc-2040, Santa Cruz Biotechnology, Santa Cruz CA) in TBS containing 1% bovine serum antigen (BSA) for 2 hours at room temperature, then incubated with primary antibodies in TBS containing 1% BSA at 4°C overnight. MIC-1 was detected with 3μg/ml goat polyclonal antibody ab39999 from Abcam (Cambridge MA; for tissues from UNMH and CHTN) or rabbit polyclonal antibody sc-66905 from Santa Cruz Biotechnology (Santa Cruz CA; for the tissue microarray). PDGF-A was detected with 3μg/ml rabbit polyclonal antibody sc-7958 from Santa Cruz Biotechnology (Santa Cruz CA). Normal rabbit IgG or normal goat IgG (10500C and 10200, respectively from Invitrogen, Carlsbad CA) was used as negative control to ensure specificity. Tissue sections were washed in TBST and incubated for 1 hour at room temperature with Alexa Fluor 633-conjugated rabbit anti-goat IgG (A21086 for MIC-1 on the UNMH/CHTN tissues) or Alexa Fluor 488-conjugated goat anti-rabbit IgG (A11008 for MIC-1 for the tissue microarrays), and with Alexa Fluor 633-conjugated goat anti-rabbit IgG (A21070 for PDGF-A) (all from Invitrogen, Carlsbad CA). All secondary antibodies were at 3.3μg/ml and in TBS containing 1% BSA and either 10% normal rabbit or 10% normal goat serum (for the antibodies raised in rabbit or goat, respectively). After washing in TBS, tissue sections were counterstained for nuclei with diamidino-2-phenylindole (DAPI; 900nM) for 2 minutes at room temperature. After washing with TBS, cells and sections were mounted in GVA Aqueous mounting medium (Genemed Biotechnologies, San Francisco CA) or Fluoroshield mounting medium (Sigma, St. Louis MO). The UNMH/CHTN tissues were analyzed by spectral image acquisition and linear unmixing performed at the University of New Mexico Health Sciences Center Fluorescence Microscopy Shared Resource Core Facility using a Zeiss LSM510 META confocal microscope with a Plan-Apochromat 63x oil 1.4 NA objective, and using lambda mode of the Zen software (Carl Zeiss MicroImaging LLC, Thornwood NY). 405 nm and 633 nm lasers were used to excite DAPI and Alexa Fluor 633, respectively, and an emission range of 433nm to 690nm was used to acquire lambda stacks and to capture the spectral information of the fluorophores. Control slides with only DAPI, only secondary antibody, and unstained tissues were imaged to acquire separate lambda stacks of each fluorescent component, i.e. DAPI, Alexa Fluor 633, and autofluorescence. Representative pixels from each of these images were selected, creating the emission spectra of each component. These spectra were then used to linearly unmix the images using the same setting in the Zen software, a process that was equally applied to all spectral images to ensure the validity of intra- and inter-tissue comparisons. Spectrally unmixed confocal images were then imported into SlideBook digital microscopy imaging software (SlideBook, Denver CO) for quantitation. The tissue microarray was analyzed by conventional immunofluorescence microscopy using a Zeiss Axiovert 35 inverted microscope equipped with a 470 excitation/525 emission Endow bandpass filter and a dichroic DAPI 360 excitation/460 emission filter. Two methods of quantitation were used: (A) Whole slide analysis (WSA); signal intensities (pixel count) were measured specifically for Alexa Fluor 633 (or Alexa Fluor 488 the tissue microarray). For the tissues from UNMH/CHTN, WSA analysis was directed specifically to cytoplasmic areas by excluding areas defined by DAPI. (B) Regions of interest analysis (ROI); 3 representative ROI (defined as areas with robust immunostaining) per slide were chosen and the signal intensities (sum pixel count per area) was determined. The size of each ROI was ~100μm^2^; for the UNMH/CHTN tissues ROI were chosen by two co-workers blinded to the nature of the tissue (F. Bisoffi and S. Jones) to avoid bias; for the tissue microarray, they were placed at equal positions across each image and the signal intensities were determined using ImageJ software (National Institutes of Health, Bethesda MD). For the images shown herein all signals (red and green) were pseudo-colored yellow for better visibility.

### Statistics

For quantitation, tissues of a kind were grouped into four different categories: (i) All combined, (ii) above and below the median to counteract the effect of heterogeneity, and (iii) non-matched to counteract the possibility of a match effect bias. Differences in expression of MIC-1 and PDGF-A between groups were analyzed by two standard statistical tests, i.e. the two-tailed student’s t-test in Microsoft Excel (Redmond WA) and, to partially control for small sample size and a distribution with infinite variance due to tissue heterogeneity, the Wilcoxon rank sums test in the JumpIn analysis software package (JumpIn Software, Cary NC). The corresponding levels of significance (p) are indicated as p(t) and p(WRS), respectively. Inter-tissue heterogeneity within a type of tissue (tumor, adjacent, or disease-free) was assessed by calculating the coefficient of variation (CV) expressed as % based on the mean of all measurements. Intra-tissue heterogeneity for the UNM/CHTN cohort was assessed by calculating the mean CV expressed as percent based of the individual CVs derived from images of individual cases. Intra-tissue heterogeneity for the tissue microarray cohort was assessed by calculating the mean CV expressed as percent based of the individual CVs derived from the ROI values of individual cases. Potential correlations of signal intensities between matched tissues were analyzed by determining the correlation coefficient (r) and confirmed by determining the independence of groups of data by the χ^2^ test. Associations with clinical parameters were analyzed using the student’s t-test. Statistical significance for all analyses was defined at p≤0.05.

## Results

### Detection of MIC-1 and PDGF-A in human prostate tissues by immunofluorescence (IF)

**Fig 1:**
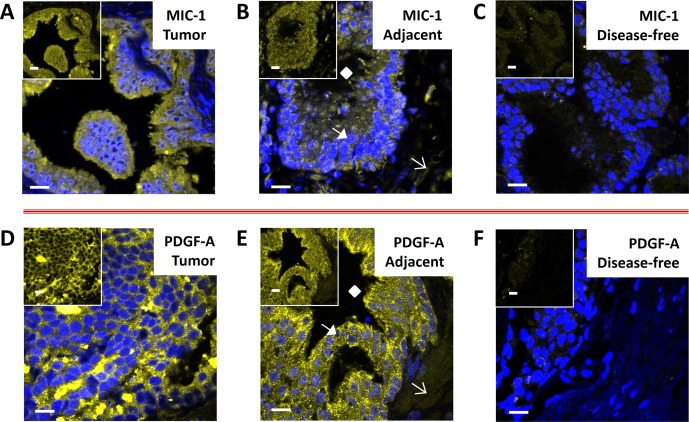
MIC-1 (A-C) and PDGF-A (D-F) detection in human prostate tissues (UNMH/CHTN cohort). Representative cases of prostate tumors (A and D) and adjacent tissues (B and E), as well as cases of disease-free control tissues unrelated to cancer (C and F) are shown; pictures represent overlays of nuclear staining by DAPI (blue) and Alexa Fluor 633 immunostaining (yellow/white); the insets are Alexa Fluor 633 immunostaining only; white bars represent 10 micrometers. The diamonds, closed arrows, and open arrows in B and E denote a typical lumen, epithelial cell compartment, and stromal cell compartment, respectively.

Upon ensuring that unspecific control IgG antibodies did not generate any measurable fluorescence, all immunostainings with specific primary antibodies were performed under identical conditions. Figs. 1A-C show representative immunostainings for MIC-1 in tumor (cancerous), tumor adjacent, and disease-free tissues, respectively. Visual inspection indicated that typically, MIC-1 expression, was similar in tumor and adjacent tissues, as well as elevated compared to disease-free prostate tissues from individuals without cancer. Although a preferential staining in epithelial compartments was observed, the staining was diffuse in appearance, seemingly both intra- and extra-cellular in nature, which is in accordance with MIC-1 being a secreted cytokine [13,14] (Figs. 1A and B). Very little, if any, MIC-1 immunostaining was observed in disease-free prostate tissues from autopsies unrelated to cancer (Fig. 1C). Likewise, immunostaining for PDGF-A was similar in tumor and adjacent tissues (Figs. 1D and E), elevated compared to disease-free tissues (Fig. 1F), and predominantly epithelial but diffuse, in agreement with a secreted growth factor [15].

### Quantitation of MIC-1 and PDGF-A expression in human prostate tissues

Using our established protocol of quantitative fluorescence based on spectral image acquisition and linear unmixing [12], a total of 190 digitized images were subjected to a comprehensive quantitation of the signal intensities representing MIC-1 and PDGF-A expression (Table 1). Differences in expression in tumor, tumor adjacent, and disease-free prostate tissues were determined in samples grouped into four different categories, i.e. (i) all combined, (ii) above and below the median to counteract the effect of heterogeneity, and (iii) non-matched to counteract the possibility of a match effect bias. These categories were analyzed using digitized images captured in WSA and ROI mode (see Materials and Methods).

**Fig 2:**
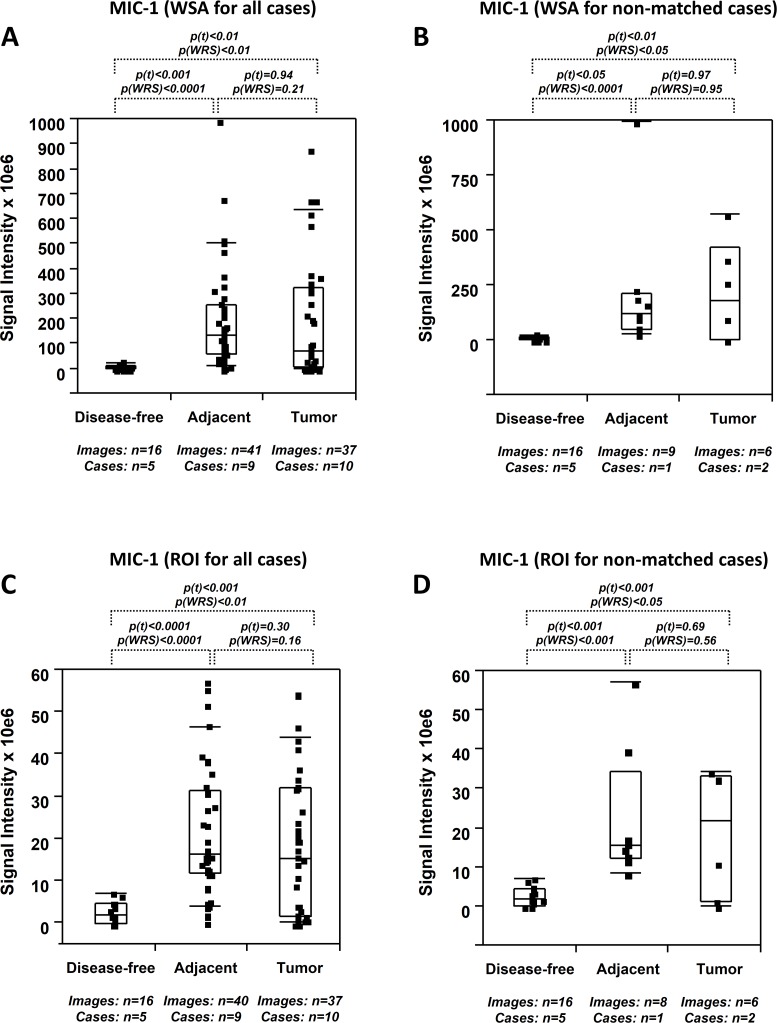
Quantitative immunofluorescence of MIC-1 in human prostate tissues. (A-D) MIC-1 expression levels (indicated as signal intensities [pixel count]) in disease-free, tumor adjacent, and tumor tissues; the types of analysis were the following (as per Materials and Methods): (A and B) Whole slide analysis (WSA) for all (A) and non-matched (B) cases in the UNMH/CHTN cohort; (C and D) region of interest (ROI) analysis for all (C) and non-matched (D) cases in the UNMH/CHTN cohort. Individual data points are shown as small black squares (partially overlapping); the boxes represent group medians (line across middle) and quartiles (25th and 75th percentiles) at its ends; lines above and below boxes indicate 10th and 90th percentiles, respectively. For each analysis, the number of images and cases is indicated; p values above the panels denote the level of statistical significance for the differences between groups, as calculated by the student’s t-test (p(t)) and by the Wilcoxon rank sums test (p(WRS)).

**Fig 3:**
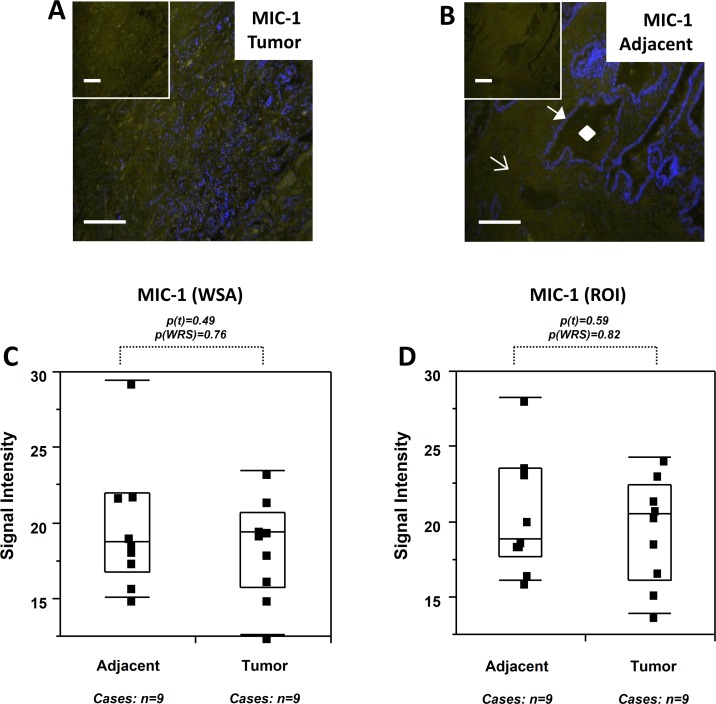
MIC-1 detection and quantitation in human prostate tissues (commercial tissue microarray). (A-B) Immunofluorescence with anti-MIC-1 antibody in a representative prostate tumor (A) and tumor adjacent tissue (B); pictures represent overlays of nuclear staining by DAPI (blue) and Alexa Fluor 488 immunostaining (yellow/white); the insets are Alexa Fluor 488 immunostaining only; white bars represent 10 micrometers. The diamond, closed arrow, and open arrow in B denote a typical lumen, epithelial cell compartment, and stromal cell compartment, respectively. (C-D) MIC-1 expression levels (indicated as signal intensities [pixel count]) in matched tumor adjacent and tumor tissues; the types of analysis were the following (as per Materials and Methods): (C) Whole slide analysis (WSA), (D) region of interest (ROI) analysis. Individual data points are shown as small black squares (partially overlapping); the boxes represent group medians (line across middle) and quartiles (25th and 75th percentiles) at its ends; lines above and below boxes indicate 10th and 90th percentiles, respectively. For each analysis, the number of images and cases is indicated; p values above the panels denote the level of statistical significance for the differences between groups, as calculated by the student’s t-test (p(t)) and by the Wilcoxon rank sums test (p(WRS)).

Expression levels for MIC-1 in tumor tissues were significantly elevated (10.2x to 23.4x by WSA and 7.1x to 9.4x by ROI) compared to disease-free tissues (p<0.05 to p<0.0001). In support of field cancerization, MIC-1 expression in tumor adjacent tissues was similarly and significantly elevated (19.2x to 38.4x by WSA and 7.4x to 20.5x by ROI) compared to disease-free tissues (p<0.05 to p<0.0001), and MIC-1 expression was similar (0.3x to 1.1x by WSA and 0.5x to 1.0x by ROI) in tumor and tumor adjacent tissues (p>0.05 for most of the analyses) (Table 2). Visual representation of data supporting prostate field cancerization for MIC-1 is given in Fig. 2. WSA analysis indicated that MIC-1 expression was significantly different between tumor and disease-free tissues (p(t)<0.01; p(WRS)<0.01) and between tumor adjacent and disease-free tissues (p(t)<0.001; p(WRS)<0.0001). In contrast, MIC-1 expression was highly similar (p(t) = 0.94; p(WRS) = 0.21) in tumor and tumor adjacent tissues (Fig. 2A). To address the possibility that matched status could influence the similarity of expression in tumor and their adjacent tissues, we determined the correlation coefficient for signal intensities derived from matched tissues, which indicated no match bias (r = 0.27). In addition, we also analyzed the difference in MIC-1 expression between images belonging to non-matched cases. Although this analysis comprised fewer data points, the difference between tumor and disease-free tissues (p(t)<0.01; p(WRS)<0.05), and between tumor adjacent and disease-free tissues (p(t)<0.05; p(WRS)<0.0001) remained significant, while MIC-1 expression in tumor and tumor-adjacent tissues (p(t) = 0.97; p(WRS) = 0.95) was similar (Fig. 2B). Figs. 2C and 2D depict similar findings for images analyzed by the ROI method. MIC-1 expression was significantly different between tumor and disease-free tissues (p(t)<0.001; p(WRS)<0.01) and between tumor adjacent and disease-free tissues (p(t)<0.001; p(WRS)<0.0001). In contrast, MIC-1 expression was similar (p(t) = 0.30; p(WRS) = 0.16) in tumor and tumor adjacent tissues (Fig. 2C). There was again no correlation in expression between matched tissues (r = 0.12) and in non-matched cases, the difference between tumor and disease-free tissues (p(t)<0.001; p(WRS)<0.05), and between tumor adjacent and disease-free tissues (p(t)<0.001; p(WRS)<0.001) remained significant, while MIC-1 expression in tumor and tumor-adjacent tissues (p(t) = 0.69; p(WRS) = 0.56) was similar (Fig. 2D). Of note, the level of inter- and intra-tissue heterogeneity for both types of measurements was high (coefficient of variation = 86.1%). Because MIC-1 expression seemed to clearly support the concept of field cancerization in prostate tissues, we determined its expression in an independent set of 9 matched tumor and adjacent tissues featured on commercially available tissue microarrays (Figs. 3A and 3B). Quantitation by both the WSA and ROI methods (Figs. 3C and 3D) revealed similar MIC-1 expression levels in tumor and tumor-adjacent tissues (0.9x by WSA [p(t) = 0.47; p(WRS) = 0.76] and 1.0x by ROI [p(t) = 0.59; p(WRS) = 0.82]) (Table 2). There was no correlation between the tumor and their adjacent tissues, indicating the absence of a match bias (r<0.1) and the inter- and intra-tissue heterogeneity was notably lower (16.9%), probably due to the conventional immunofluorescence method.

**Table 2:** Differences in expression of MIC-1 and PDGF-A in tumor, tumor adjacent, and disease-free prostate tissues obtained from UNMH/CHTN and on tissue microarrays (TMA).

			Tumor: Disease-free		Adjacent: Disease-free		Tumor: Adjacent	
			Ratio ^1^	Significance (p) ^2^	Ratio	Significance (p)	Ratio	Significance (p)
**MIC-1**	**WSA** ^**3**^	***All***	20.1	<0.01; <0.01	20.5	<0.001; <0.0001	1.0	= 0.94; = 0.21
*UNMH*		***Above median***	21.5	<0.001; <0.0001	19.2	<0.001; <0.0001	1.1	= 0.59; = 0.48
*CHTN*		***Below median***	10.2	= 0.08; <0.05	38.4	<0.001; <0.0001	0.3	<0.001; <0.001
		***Non-matched***	23.4	<0.01; <0.05	22.9	<0.05; <0.0001	1.0	= 0.97; = 0.95
	**ROI** ^**4**^	***All***	7.1	<0.001; <0.01	8.6	<0.0001; <0.0001	0.8	= 0.30; = 0.16
		***Above median***	7.4	<0.0001; <0.0001	7.4	<0.0001; <0.0001	1.0	= 0.98; = 0.93
		***Below median***	9.4	= 0.05; <0.01	20.5	<0.0001; <0.0001	0.5	<0.01; <0.01
		***Non-matched***	7.6	<0.001; <0.05	9.1	<0.001; <0.001	0.8	= 0.69; = 0.56
**MIC-1**	**WSA**		n/a ^5^	n/a	n/a	n/a	0.9	= 0.47; = 0.76
*TMA*	**ROI**		n/a	n/a	n/a	n/a	1.0	= 0.59; = 0.82
**PDGF-A**	**WSA**	***All***	2.7	= 0.13; = 0.15	2.1	= 0.23; = 0.34	1.3	= 0.33; = 0.47
*UNMH*		***Above median***	2.8	= 0.06; <0.05	2.2	= 0.12; = 0.13	1.3	= 0.25; = 0.19
*CHTN*		***Below median***	2.1	= 0.23; = 0.16	1.7	= 0.23; = 0.20	1.2	= 0.36; = 0.81
		***Non-matched***	3.7	= 0.05; <0.05	1.4	= 0.51; = 0.50	2.6	<0.05; = 0.07
	**ROI**	***All***	1.8	= 0.09; = 0.10	1.9	= 0.11; = 0.10	1.0	= 0.90; = 1.00
		***Above median***	1.7	<0.05; <0.05	1.8	<0.05; = 0.05	0.9	= 0.65; = 0.64
		***Below median***	3.0	<0.05; <0.05	2.7	<0.05; <0.05	1.1	= 0.61; = 0.66
		***Non-matched***	3.1	<0.01; <0.01	1.6	= 0.35; = 0.19	1.9	<0.01; = 0.05

Tissues of a kind were grouped into four different categories, i.e. (i) all combined, (ii) above and below the median to counteract the effect of heterogeneity, and (iii) non-matched to counteract the possibility of a match effect bias.

**Fig 4:**
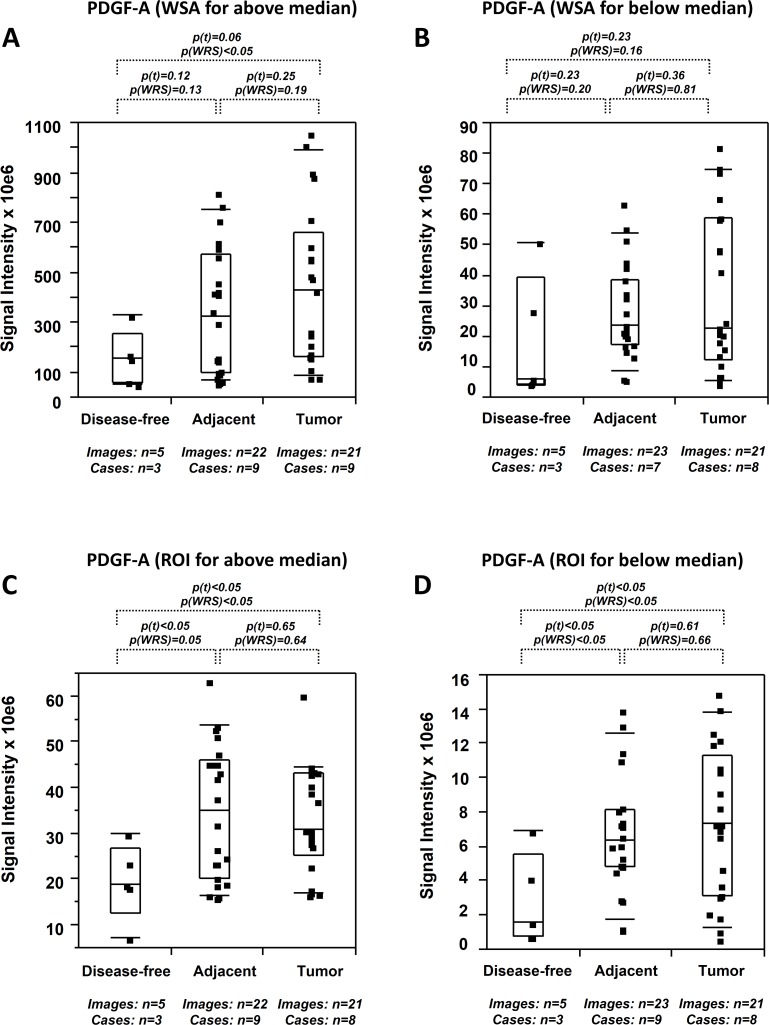
Quantitative immunofluorescence of PDGF-A in human prostate tissues. (A-D) PDGF-A expression levels (indicated as signal intensities [pixel count]) in disease-free, tumor adjacent, and tumor tissues; the types of analysis were the following (as per Materials and Methods): (A and B) Whole slide analysis (WSA) for values above (A) and below (B) the median of the values of all cases in the UNMH/CHTN cohort; (C and D) region of interest (ROI) analysis for values above (A) and below (B) the median of the values of all cases in the UNMH/CHTN cohort. Individual data points are shown as small black squares (partially overlapping); the boxes represent group medians (line across middle) and quartiles (25th and 75th percentiles) at its ends; lines above and below boxes indicate 10th and 90th percentiles, respectively. For each analysis, the number of images and cases is indicated; p values above the panels denote the level of statistical significance for the differences between groups, as calculated by the student’s t-test (p(t)) and by the Wilcoxon rank sums test (p(WRS)).

^1^ Ratio is based on the mean of all pixel intensities (expression) in the indicated group. The number of images utilized to calculate the ratio and the difference in expression are partially indicated in Table 1 and Figs. 2 and 4.

^2^ First significance measure p for the difference in expression is from the student’s t-test; the second significance measure p is from the Wilcoxon rank sums test.

^3^ WSA, analysis by whole slide.

^4^ ROI, analysis by region of interest.

^5^ n/a, not applicable.

PDGF-A expression in tumor tissues was elevated to a lesser extent and less robustly than MIC-1 (2.1x to 3.7x by WSA and 1.7x to 3.1x by ROI) compared to disease-free tissues (p<0.01 to p = 0.23). Its expression in tumor adjacent tissues was slightly and less robustly elevated (1.4x to 2.2x by WSA and 1.6x to 2.7x by ROI) compared to disease-free tissues (p<0.05 to p = 0.51) (Table 2). PDGF-A expression was analyzed as a function of the data median (Fig. 4). Accordingly, WSA analysis indicated that PDGF-A expression was significantly different between tumor and disease-free tissues (p(t) = 0.06; p(WRS)<0.05) but not between tumor adjacent and disease-free tissues (p(t) = 0.12; p(WRS) = 0.13) for expression levels above the median (Fig. 4A). Expression levels below the median did not reveal any distinction between these types of tissues (p = 0.16 to p = 0.23) (Fig. 4B). Analysis by the ROI method revealed a more supportive picture for field cancerization. PDGF-A expression was significantly different for both data sets above and below the mean between tumor and disease-free tissues (p(t)<0.05; p(WRS)<0.05) and between tumor adjacent and disease-free tissues (p(t)<0.05; p(WRS)≤0.05), while expression was similar in tumor and tumor adjacent tissues (p(t) = 0.61 to 0.65; p(WRS) = 0.64 to 0.66) (Figs. 4C and 4D and Table 2). Similar to MIC-1, the level of inter- and intra-tissue heterogeneity for all types of measurements was high (coefficient of variation = 85.9%).

## Discussion

Field cancerization in prostate tissues is well recognized, either as a state of molecular premalignancy preceding histological change or as a reaction to the presence of a tumor within the gland. This intriguing concept includes a multitude of cells and cellular functions, and a growing list of characterizing factors [7–10,16]. We have previously reported on telomere length, a marker of genomic instability [17,18], and have more recently focused on the deregulation of expression of protein factors [11,12]. The present study represents a contribution to this line of research and adds two secreted protein factors to the list of markers and/or potential mediators of field cancerization, i.e. MIC-1 and PDGF-A. Previous reports by us and others showed their deregulation at the transcriptional level without information on protein expression [11,19,20]. In contrast, we provide here a detailed quantitation of the corresponding protein expressions in human prostate tissues.

MIC-1 and PDGF-A are secreted factors with an established role in prostate tumorigenesis and cancer progression [21–25]. The role of MIC-1 is less clear and is reported as both a cancer promoter and suppressor [13,25,26]. Interestingly, MIC-1 was first discovered in macrophages [27], but when secreted by prostate cancer cells, it may promote a pro-tumorigenic environment by suppressing the anti-cancer activity of immune cells [23]. The role of PDGF-A in prostate cancer is more established. PDGF-A is one of four isoforms that form functional dimers and bind to the tyrosine kinase receptors PDGFRα and PDGFRβ. PDGFs stimulate growth, survival, and motility of various cell types and have important functions during embryonic development and in the control of tissue homeostasis. Hyperactive PDGF signaling is associated with prostate cancer development and progression through paracrine and autocrine actions [21,22], and constitutes a major molecular target for clinically established therapeutic agents such as imatinib [28]. Secreted, as opposed to intra-cellular, markers and mediators of field cancerization fit two major models to explain the development of molecularly altered fields [9]. Accordingly, cells with up-regulated MIC-1 and PDGF-A expression could act locally in an autocrine manner, inducing small hyperproliferative cell consortiums prone to further genetic and biochemical change towards transformation. Alternatively, when secreted by cancer cells, they could influence more distant areas of the prostatic gland, thereby inducing secondary fields of molecularly altered cells and perhaps inducing the well described multifocality of prostate cancer [29].

With respect to biomedical research, our observations support previous notions that tissues adjacent to tumors may not represent the most ideal controls for molecular studies [30]. Further, we and others have previously argued that markers of field cancerization may have potential clinical applications [7–10]. A potential limitation is the heterogeneity featured among and even within tissues. This could be due in part to the sophisticated and sensitive quantitation method utilized in this study and should not bar the definition of clinically informative threshold values in future studies. Although the present study was not designed nor powered to conclusively determine the association of MIC-1 and PDGF-A with clinical parameters, we observed that MIC-1 expression levels significantly differentiated pathological stage T2 from T3 in tumor tissues (p<0.001 to p<0.05), and were moderately associated with Gleason scores ≤6 *vs*. 6 and ≤7 *vs*. 7 (p<0.05 to 0.13). This is in agreement with the previously reported capacity of MIC-1 to act as a prognostic marker in prostate cancer [31–33] and corroborates our staining and quantitation approach. In contrast, MIC-1 expression in tumor adjacent tissues did not show any association with pathological stage or Gleason grade. An association of PDGF-A expression with clinical parameters was less obvious, but it is noteworthy that in both tumor and tumor adjacent tissues, PDGF-A expression tended to differentiate between Gleason scores ≤6 *vs*. 6 (p<0.05 to 0.13). Although the PDGF / PDGFR signaling pathway is strongly implicated in the development and progression of prostate cancer [21,22], its capacity as independent prognostic biomarker in prostate cancer has not been thoroughly described. Our work indicates that protein markers of field cancerization may be more exploitable as diagnostic rather than prognostic markers [12]. They could increase the clinically informative area surrounding biopsy cores, thereby indicating the presence of a tumor or providing a target for repeated biopsy in first false negative cores from patients with suspected prostate cancer following elevated PSA and/or abnormal digital rectal examination (DRE). An improved biopsy strategy would minimize potential side effects, such as hematuria and hematospermia [34]. Clinical studies could determine whether elevated expression of secreted factors at the inked margin of prostatectomy specimens predicts extra-capsular extension. Such studies would add molecular information towards a more refined definition of surgical margins, potentially providing complementary information improving clinical decision-making. Such markers could further be indicators of efficacy of neo-adjuvant drugs applied in pre-surgical therapeutic interventions, and guiding markers for focal therapy regimens [7–10]. Finally, if causative for cancer development, they could represent molecular targets for prevention or used to help identify men at higher than normal risk for developing cancer. Selective chemoprevention in these men might prove more cost-effective than treating larger populations [35].
